# Meglumine antimoniate intralesional infiltration for localised cutaneous leishmaniasis: a single arm, open label, phase II clinical trial

**DOI:** 10.1590/0074-02760180200

**Published:** 2018-06-21

**Authors:** Dario Brock Ramalho, Rosiana Estéfane da Silva, Maria Camilo Ribeiro de Senna, Hugo Silva Assis Moreira, Mariana Junqueira Pedras, Daniel Moreira de Avelar, Lara Saraiva, Ana Rabello, Gláucia Cota

**Affiliations:** 1 Fundação Oswaldo Cruz, Instituto René Rachou, Pesquisa Clínica e Políticas Públicas em Doenças Infecto-Parasitárias, Belo Horizonte, MG, Brasil; 2 Fundação Hospitalar do Estado de Minas Gerais, Hospital Eduardo de Menezes, Belo Horizonte, MG, Brasil

**Keywords:** intralesional infiltration, meglumine antimoniate, cutaneous leishmaniasis, therapy

## Abstract

**BACKGROUND:**

Cutaneous leishmaniasis (CL) is a world-wide health problem which currently lacks effective, affordable and easy to use therapy. Recently, the meglumine antimoniate (MA) intralesional infiltration was included among the acceptable therapies for New World leishmaniasis. While this approach is attractive, there is currently little evidence to support its use in Americas.

**OBJECTIVES:**

The aim of this study was to provide information about effectiveness and safety of a standardised MA intralesional infiltration technique for the treatment of CL.

**METHODS:**

It is a single-arm phase II clinical trial conducted at a Brazilian referral centre. CL cases with parasitological confirmation presenting a maximum of three CL-compatible skin lesions were treated with weekly MA intralesional infiltration by using a validated technique, up to a maximum of eight infiltrations.

**RESULTS:**

A total of 53 patients (62 lesions) were included. Overall, patients received a median of seven infiltrations (IQR25-75% 5-8) over a median treatment period of 43 days (IQR25-75% 28-52 days). The definitive cure rate at D180 was 87% (95% CI:77-96%). The majority of adverse events were local, with mild or moderate intensity. Bacterial secondary infection of the lesion site was observed in 13% of the treated patients, beside two intensity-three adverse events (hypersensitivity reactions).

Cutaneous leishmaniasis (CL) is a non-fatal yet severely disfiguring condition that progresses over weeks to months and has a broad spectrum of manifestations including papules, nodules, plaques and ulcerative lesions, with the latter being the most common form in Americas, where the spontaneous cure rate is very low.^1^ Over the past decade, the worldwide prevalence and geographical distribution of CL has expanded as a result of the dynamic evolution of the transmission foci.^2^ The condition is recognised as being a complex and highly variable disease in terms of epidemiology, aetiology, pathology, and clinical features.

The mainstays of treatment for CL around the world are pentavalent antimony compounds administered parentally. The risk of developing mucosal leishmaniasis (ML), a feared complication due to *Leishmania braziliensis*, is the main reason for the historic use of systemic treatments. It is a paradigm that has been recently questioned since this metastatic complication has never been systematically studied, and there are no reports on mucosal complications developing in CL patients who were treated with topical therapies.^3^ Topical and local treatments could offer significant advantages such as an easier administration, less adverse effects and lower costs, contrasting clearly with the well-described toxic complications with the systemic use of antimony derivatives. Intralesional treatments are used on a regular basis in Old World CL but not in New World disease^4^, although it already has been recommended by the World Health Organization Expert Committee on Leishmaniasis^5^ and Pan American Health Organization (PAHO).^6^ The aim of the present study was to assess the efficacy of a standardised regimen of MA intralesional infiltration for CL treatment.

## SUBJECTS AND METHODS

This study is a single arm, phase II, clinical trial conducted at a referral centre for CL treatment: Instituto René Rachou, Fundação Oswaldo Cruz (IRR-FIOCRUZ), Minas Gerais (MG), Brazil. Patients presenting with up to three active cutaneous lesions and with parasitological confirmation of *Leishmania* infection were eligible for inclusion.

*Ethical aspects and study registration* - The study (CAAE 44674314.3.3001.5091) was approved by the Research Ethics Committee of the Instituto René Rachou Research (number 1,136,132). This trial is registered with Brazilian Registries of Clinical Trials (REBEC) under number RBR-44KG5X, and has Universal Trial Number (UTN) U1111-1171-8847. Patients consent was obtained in writing.

*Recruitment, inclusion criteria and exclusion criteria* - All of the patients consecutively seen at the first appointment who had suspicion of CL between August 1, 2015 and January 30, 2017 were enrolled and evaluated according to the following inclusion and exclusion criteria:

*Inclusion criteria* - (i) The presence of a maximum of three CL - compatible skin lesions not involving ear, mucosal or joint regions and with a total area < = 900 mm^2^ (according to Olliaro et al. ^7^ and considering all lesions); (ii) the parasitological confirmation of *Leishmania* spp. infection by direct examination (imprint smear), culture, histological examination or kinetoplast DNA based polymerase chain reaction (PCR).

*Exclusion criteria* - (i) Less than 13 years old; (ii) previous treatment with anti-*Leishmania* active drugs in the six months prior to enrolment; (iii) a history of MA allergy; (iv) current pregnancy or lactation; (v) mucosal involvement; (vi) the presence of a significant clinical condition that at the discretion of the investigator contraindicates the use of MA, including cardiac, hepatic or renal dysfunction.

**Fig. 1:**
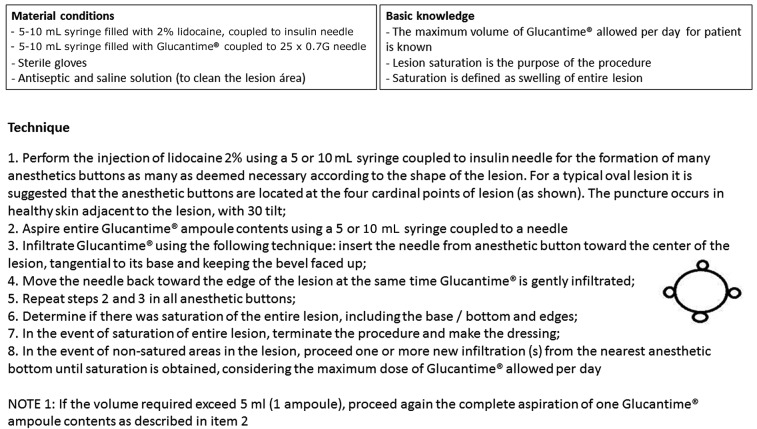
the standard operational procedure for the meglumine antimonial intralesional infiltration technique.

*Intervention* - The experimental therapeutic intervention consisted of weekly (7 ± 3 days) intralesional infiltration of MA following a previously validated standardised technique^8^ (Fig. 1). Treatments continued until complete wound healing or a maximum of eight infiltrations occurred. Intralesional infiltration was postponed 7-14 days if bacterial infection was identified at the lesion. All of the infiltrations were performed with the same lot of Glucantime^®^ control number (528240), which was produced by Sanofi-Aventis and assigned by the Brazilian Ministry of Health. The medication is packaged in 5 mL ampoules with a concentration of 81 mg/mL pentavalent antimony (Sb^v^).

Prior to the infiltration, the lesions were cleaned with sterile 0.9% saline and chlorhexidine and the scabs were removed. At first, local anaesthesia using 2% lidocaine was performed from strategic points located in the uninjured skin adjacent to the lesion, according to the instructions contained in the standard operational procedure (SOP). The total volume of the Glucantime^®^ ampoule was aspirated (5 mL) with a 5 mL syringe and the intralesional infiltration was performed with a 25 mm x 0.8 mm needle, from the anaesthetic button towards the centre of the lesion, with a depth of 1 mm to 2 mm inside the skin, and with the needle bevel facing upward and an inclination of the needle as parallel as possible to the plane of the lesion surface. The infiltration was repeated as many times as necessary, on radial route, until reaching the lesion state defined as “saturation” was reached or until a volume of 15 mL (or the volume corresponding to 20 mg/kg of MA - whichever occurred first) was applied.

Lesion saturation was defined as the total swelling or pallor of the skin lesion, including the borders and centre. Alternatively, saturation was also defined by extravasation of the medication in two attempts at infiltration, even if the swelling and pallor had not been achieved. The total volume applied during each infiltration session was recorded at the end of the procedure.

Considering the day of the first intralesional infiltration as D1, clinical and laboratory monitoring was performed weekly during the treatment period and at the D90 and D180 visits after that. In all of the visits, a questionnaire including information on the occurrence of adverse events and on the physical examination, which included vital signs, patient weight, mensuration and photographic documentation of skin lesions was performed. Laboratory tests were also performed weekly, including electrocardiogram, haemogram and the following biochemical dosages: amylase, lipase, bilirubin, urea, creatinine, oxalacetic and pyruvic transaminases.

All of the identified adverse events were classified using a four intensity-grade system classification^9^ (ACTG - Division of AIDS - DAIDS, 2014) The causal relationship between the event and the treatment was evaluated according to the classification system recommended by the World Health Organization (WHO-UMC).^10^

*Definitions of endpoints* - According to the current recommendations for the standardisation of the methodology applied to clinical trials on CL^7^, the following cure criteria were adopted:

*Initial cure* - Evaluated at 90 days ± 14 days from the start of the treatment (D90), defined by complete epithelisation of all the ulcers, complete involution of the nodule or plaque and the absence of any signs of inflammatory activity.

*Definitive cure* - Evaluated at 180 days ± 14 days after initiation of the treatment (D180), defined by complete epithelisation of all ulcers, complete involution of the nodules or plaques and the absence of any signs of inflammatory activity.

*Failure* - Defined as non-reduction of at least 50% of the lesion area at 45 ± 7 days after initiation of treatment (D45), also any condition other than a cure at D90 or D180 or the appearance of any new lesion after the initiation of treatment.

*Criteria for suspension of the treatment* - The treatment with intralesional infiltration of MA was definitively discontinued in the following situations:

(i) Occurrence of a severe adverse event (graded as four), provided the causal relationship between the event and the treatment (probable or confirmed according to WHO-UMC system)^10^;

(ii) More than 21 days since the last infiltration (the maximum infiltration interval allowed was 21 days);

(iii) Complete healing of the lesion;

(iv) Completion of eight intralesional infiltrations;

(v) Withdrawal of consent by the patient.

*Statistical analysis* - The descriptive analysis included simple frequencies and the median and its respective interquartile range of 25-75% (IQR 25-75%) or the mean and standard deviation, whenever appropriate. The continuous variables were analysed using unpaired Student’s *t tests* for normally distributed variables and Wilcoxon tests for variables with skewed distributions. Chi-square tests were used to compare categorical variables. Univariate analyses for factors associated with treatment failure at six months were performed. Statistical significance was set at the 0.05 level. All of the analyses were performed using Statistical Product and Service Solutions, IBM - SPSS^®^ (version 16, California, USA).

## RESULTS

**Fig. 2:**
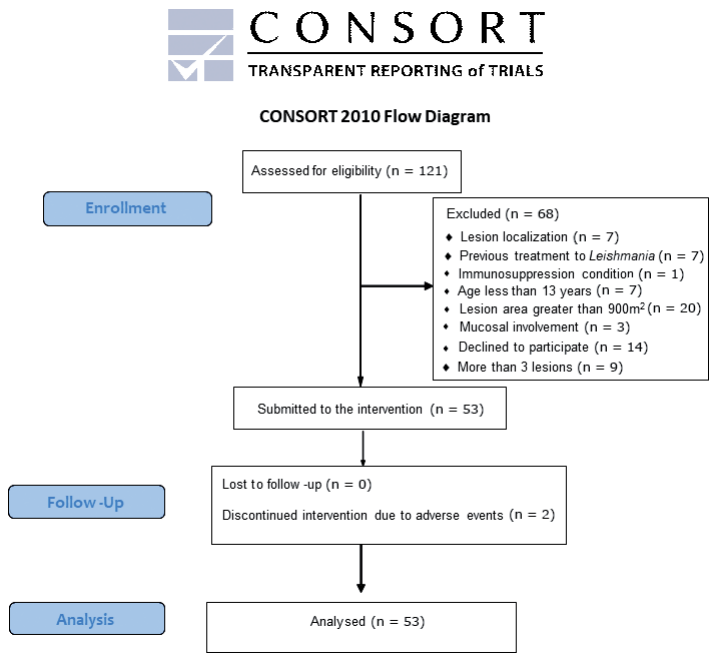
study flowchart according CONSORT stands.

During the 18-month study period, one hundred twenty-one patients were diagnosed with CL at the referral centre. Sixty-eight patients could not be included in the study due to the following reasons: an immunosuppression condition (one patient with ankylosing spondylitis), an age less than 13 years (seven patients), a lesion area with an area greater than 900 mm^2^ (20 patients), more than three lesions (nine patients), mucosal involvement (three patients), previous treatment against *Leishmania* (seven patients), refusal by the patient (14 patients), and a lesion located on the ear or joint region (seven patients). The flowchart of patients included, treated and evaluated, according to the CONSORT stands for Consolidated Standards of Reporting Trials, is shown in Fig. 2.

A total of 53 patients (32 men and 21 women) were included in the study protocol. The age of the patients ranged from 14 to 75 years (median, 42) years. The median duration of lesions prior to treatment was 10 weeks (IQR 25-75% 8 to 16) weeks. The total number of lesions was 62 and they were distributed in the limbs (79%), trunk (13%), and head (8%). Fifty-seven of the 62 lesions (92%) were ulcered and five of the lesions (8%) were nodules. The demographic and clinical characteristics of the study patients are presented in Table I.

**TABLE I:** Demographical and clinical characteristics of cutaneous leishmaniasis (CL) patients treated with meglumine antimoniate (MA) intralesional infiltration in a Brazilian referral centre

Characteristics	53 patients, 62 lesions
Age (median, IQR), years	42 (31-58)
Sex (male: female)	29:17
Lesion location, No. (%)	
Lower limbs	25/62 (40)
Arms/hand	24/62 (39)
Trunk	8/62 (13)
Head/neck	5/62 (8)
Number of lesions per patient (mean, SD)	1.17 ± 0.47
Feature of the main lesion: ulcerated, n (%)	49/62 (92)
Site of the main lesion: lower limbs, n (%)	23/62 (43)
Total lesions area (Median, IQR), mm^2^	462 (212-703)
Diameter of the main lesion (Median, IQR), mm	25 (16.5-31)
Montenegro skin test positivity, n/N (%)	19/22 (86)
Imprint smear positive, n/N (%)	33/44 (75)
*Leishmania* culture positive, n/N (%)	37/48 (77)
Positive *Leishmania* PCR, n/N (%)	7/7 (100)
Illness duration before treatment (median, IQR), weeks	10 (8-16)
Hypertension, n (%)	13/53 (24)
Diabetes, n (%)	1/53 (2)

IQR: 25-75% interquartile range; SD: standard deviation; N: number of patients tested; PCR: polymerase chain reaction.

All 53 of the patients completed the proposed treatment and the post-treatment visits, with none lost to follow-up. The median total time of treatment was 43 days (IQR 25-75% 28-52) days with a median number of infiltrations of 7 (IQR 25-75% 5-8). The median total volume of Glucantime^®^ infiltrated per patient during entire treatment period was 27.7 mL (IQR 25-75%: 16.4-46.4) mL. Thus, this volume distributed in seven infiltrations corresponds to approximately 3.9 mL of Glucantime^®^ infiltrated per week, which is equivalent to 315.9 mg of Sb^v^ every seven days. According to the protocol, the interval between the infiltrations should be 7 ± 3 days. The observed mean of the intervals between the applications was 7 ± 2 days, ranging from five to 21 days due to the presence of bacterial infection in the site of the lesion or non-attendance to the scheduled appointment (patient absence, holidays, etc.). In only three occasions (three patients) it was observed an interval higher than 10 days. The treatment details and outcomes are shown in Table II.

**TABLE II:** Summary of the antimony intralesional infiltration therapy

Number of infiltrations (median, IQR)	7 (5-8)
Total volume of Glucantime^®^ infiltrated (median, IQR), mL	27.7 (16.4-46.4)
Total antimony dose infiltrated (median, IQR), mg	2243 (1328-3.758)
Treatment duration - days between the first and the last infiltration (median, IQR), days	43 (28-52)
Time until cure (median, IQR), weeks	7 (6-13)
Interval between infiltrations (mean, SD), days	7.6 ± 2.2
Lost to follow-up, n (%)	0 (0)
Adverse events	
Local bacterial infection, n (%)	7 (13.2)
Severe adverse event, n (%)	1 (1.9)
Local irritation (erythema/oedema/itching), n (%)	53 (100)
Local vesicles/bullae, n (%)	2 (3.7)
Outcomes	
Cured patients at the 45-day visit, n (rate, 95%CI)	32 (60%, 7-72%)
Cured patients at three months, n (%)	46 (87%, 75-94%
Cured patients at six months, n (%)	46 (87%, 75-94%)
Relapse at six months	1/46 (2.2)

IQR: 25-75% interquartile range; SD: standard deviation; 95% CI: 95% confidence interval.

At the 45-day visit, three of the 53 patients (6%) presented with lesions with an area equal or greater than to 50% of the initial size. They were considered as being therapeutic failures and underwent rescue therapy with MA by the parenteral route which resulted in subsequent healing of their lesions.

At the 90-day visit, four more patients were considered as being therapeutic failures. Of these four patients, one patient had a crusted area of approximately 3 mm, which was interpreted as a residual ulcer. This patient received an additional MA intralesional infiltration and the lesion was considered to be completely healed approximately 20 days later. Another one of these patients presented with a new skin ulcer. He underwent therapy with parenteral MA and was also cured. The other two patients had complete epithelisation of their ulcers but local skin infiltration could be still observed. One of them was considered to be cured at D180 without further treatment, and another, for whom the infiltrations persisted, was referred to treatment with parenteral MA and was cured.

**Fig. 3:**
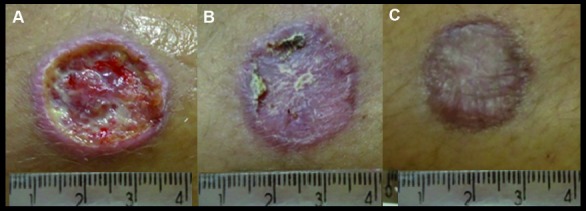
(A) The first day of the treatment (D1); (B) 42 days from the beginning of the treatment (D42); (C) 90 days from the beginning of the treatment (D90).

At six months of follow-up, in addition to the patient who maintained persistent infiltration in the lesion since the D90-visit, another patient, whose lesion was considered to be inactive at the D90-visit, was considered to be a therapeutic failure when the presence of lesion skin infiltration was detected at the D180-visit. She was submitted to parenteral MA salvage therapy and was cured. Thus, the definitive cure rate observed was 87% (95%CI 75-94) %. Analysing only the 49 patients who had exclusively ulcerated lesions, the evolution towards complete epithelisation and disappearance of local inflammatory signs over time are shown in Table III. An illustration of the evolution of a leishmaniasis skin lesion treated with MA intralesional infiltration is shown in Fig. 3.

The exploration of factors associated with a cure was performed by a univariate analysis (Table IV). Analysis of demographic, clinical and therapy-related characteristics failed to reveal an individual risk factor for treatment failure at 180 days.

The majority of adverse events were localised to the injection site, and included pruritus, oedema, pain and redness. In all of the cases, local events were of a mild or moderate intensity. No new laboratory or electrocardiogram abnormality was observed after the beginning of the treatment. Seven of the 53 patients (13%) presented with bacterial secondary infections of the lesion site. All seven successfully treated with oral antibiotic for seven days. Two patients (4%) had intensity three adverse events: a local delayed type IV hypersensitivity reaction with hypermia, oedema and blisters, twice, hours after the third and fourth intralesional infiltration sessions; the other, a systemic type I hypersensitivity reaction with hypotension during the fourth intralesional infiltration (28 days after the first infiltration), suggesting that re-exposure to the stimulus amplified the reaction. The both patients had the intralesional treatment withdrawn, without any additional treatment for CL. They had complete involution of the signs and symptoms, despite the premature suspension of MA treatment, and were considered to be cured at D180. A detailed description of the adverse events observed and their correlation with the serum antimony dosage will be shown in another manuscript in the future.

**TABLE III:** The clinical evolution of the 49 patients who presented with exclusively ulcered lesions throughout the treatment duration

	Complete re-epithelisation n (%)	Complete re-epithelisation with no inflammatory signs n (%)
Second week (one infiltration)	6 (12)	0 (0)
Third week (two infiltrations)	11 (22)	3 (6)
Fourth week (three infiltrations)	12 (24)	7 (14)
Fifth week (four infiltrations)	16 (33)	8 (16)
Sixth week (five infiltrations)	21 (43)	14 (28)
Seventh week (six infiltrations)	27 (55)	21 (42)
Eighth week (seven infiltrations)	34 (69)	27 (55)

**TABLE IV:** The characteristics of the patients at the 180-day visit (from the univariate analysis)

	Cured patients (%) n = 46	Not cured patients (%) n = 7
Age (median, IQR), years	42.5 (29.7-59.7)	42 (33-53)
Sex (male: female)	29:17	3:4
Lesion location, No. (%)		
Lower limbs	22 (47.8)	3 (42.8)
Arms/hand	22 (47.8)	2 (28.6)
Trunk	8 (17.4)	0
Head/neck	3(6.5)	2 (28.6)
Patients with more than one lesion	7 (15.2)	0
Feature of the main lesion - ulcerated, n (%)	42 (91.3)	7 (100)
Site of the main lesion - lower limbs, n (%)	20 (43.5)	3 (42.8)
Total lesions area (median, IQR), mm^2^	465 (214.2-709.2)	390 (156-702)
Patient weight	67 (59.8-81.1)	68.2 (53.3-82.2)
Diameter of the main lesion (mean, SD), mm	23.5 ± 8.9	27.3 ± 9
Montenegro skin test positivity, n/N (%)	16/17 (94.1)	3/4 (75)
Imprint smear positive, n/N (%)	30/40 (75)	3/4 (75)
Positive *Leishmania* culture, n/N (%)	32/42 (76.1)	5/6 (83.3)
Positive *Leishmania* PCR, n/N (%)	6/46 (13)	1/7 (14.2)
Illness duration (median, IQR), weeks	12 (8-16)	9 (7-12)
Hypertension, n (%)	10/46 (21.7)	3/7 (42.8)
Diabetes, n (%)	1 (2.1)	0
Number of infiltrations (median, IQR)	6.5 (4-8)	7 (5-8)
Total volume of Glucantime^®^ infiltrated (mean, SD), mL	55.1 ± 92	32 ± 17
Total antimony dose infiltered (median, IQR), mg	2324 (1215-3709)	1984 (1782-4131)

IQR: 25-75% interquartile range; SD: standard deviation.

## DISCUSSION

Historically, antimonial drugs, either intravenously or intramuscularly, have been widely used for CL treatment in the Americas despite their recognised toxicity. This reality is still regarded as acceptable in the face of the inexistence of another effective and affordable therapy. In contrast, the localised use of antimony derivatives has been described at least for three decades and is mainly used in the treatment of Old World CL.^4^ Recently, intralesional therapy has been included among the recommendations for New World CL treatment^5^ ^,^ ^6^, despite the scarcity of evidence showing effectiveness for CL in Americas.^11^ ^-^ ^15^ The potential advantages of the intralesional infiltration are the use of lower total doses of antimony, which in theory, could reduce its toxicity and would allow for a more flexible schedule.

Two studies in Bolivia have addressed the use of antimony intralesional therapy in New World leishmaniasis.^16^ ^,^ ^17^ These studies are limited by the number of infiltrations and a short interval between them (1-5 days). The current study, using a standard approach and a greater number of infiltrations was associated with an effectiveness of 87%. It should be noted that, according to a systematic review gathering the available options for New World CL treatment^18^, pentavalent antimonial derivatives, which are the most studied drugs, promote a 76.5% cure rate. The efficacy of pentamidine was similar to that of these derivatives, while other drugs showed variable results, but always an inferior response. Similarly, the cure rate with pentamidine intralesional was recently determined^17^ as being 72% and oral miltefosina was considered to be statistically not different than antimony.^19^ In addition, direct comparisons of antimony intralesional infiltration with topical 15% paromomycin ointment, radiofrequency-induced heat therapy, topical trichloroacetic acid and cryotherapy showed no significant difference in efficacy between the interventions, according to recent systematic review^4^, all studies performed in the Middle East and India. All of these findings confirming the lack of a therapeutic option significantly superior to the others, in terms of effectiveness, until the present day.

In general, the cure rate here observed can be considered similar to other previously obtained for the MA intralesional approach in the Americas. Also based on the same systematic review^4^, seven studies addressing intralesional infiltration for New World CL are available. These studies involved 512 participants and had an overall efficacy of 76.9% (95%CI 66-85%, I^2^ = 69.5). Three studies (230 patients) were randomised trials conducted by the same investigator^16^ ^,^ ^17^ but compared different treatment arms. The efficacy was higher in the non-randomised studies (282 patients) than in the randomised studies: 84.6% (95%CI 72.2-92.1%) *versus* 63.2% (95%CI 52.8-72.6%), p = 0.00. In Bolivia, Soto and colleagues^16^ have proposed smaller intervals and a small number of infiltrations (three or five), which had efficacy rates of 70% and 73%, respectively.

It should be noted that the MA intralesional therapy would not be a suitable alternative for all cases of CL, but rather for patients with few cutaneous lesions, considering the nature of the procedure, which requires the drug to be infiltrated into each one of the lesions. Even accepting this limitation, the wide application of the intralesional approach would be significant, since patients with single or few skin lesions represent more than 70% of the total CL cases in the Americas^20^. It is also important to mention another potential problem inherent to the intralesional approach: the requirement of a medical doctor to perform the therapeutic procedure. However, in view of the even greater toxicity related to the parenteral antimony use, which also requires medical monitoring and intervention, this aspect cannot be considered as a disadvantage in comparison with the most commonly used therapy today.

Another unanswered question is regarding the risk for late mucosal complications related to non-systemic treatments, which is a theoretical concern comprising for not only intralesional infiltration but also thermotherapy, cryotherapy and other topical therapies. It has been the main reason for the recommendation of systemic treatment for all CL patients in the New World, a paradigm only recently challenged, and responsible for the delay in the study of topical approaches for CL in the Americas and, perhaps also for hundreds of deaths each year due to antimony toxicity. The association between local treatments and the risk of ML (compared to systemic treatment) has never been systematically studied, and there are no reports on ML developing in New World CL patients who were treated with intralesional or other local approaches^3^ ^,^ ^12^ ^,^ ^15^. At this stage of this phase II study, only the short-term follow-up data is presented, which may be considered as one of its limitations. However, all of the treated patients are undergoing follow-up and were advised about the risk for relapse. Thus, in the future, long-term outcome data may be presented.

Another limitation that should be noted is the lack of characterisation of the parasite involved in the infection of the treated patients, since there is growing evidence on the the great intraspecific genetic heterogeneity related to susceptibility pattern among *L. braziliensis* species^21^, which may justify part of the clinical response, and limit the extrapolation of our findings to other regions.

On the other hand, for the intralesional therapeutic modality, one of the main difficulties for comparing different studies is the lack of standardisation of the procedure, not only for the therapeutic regimen but the technique itself. Facing the few detailed descriptions available for the technique in the literature, we recently published a standardisation of the intralesional infiltration procedure and its validation, the protocol that was followed in this study^8^. Even so, one of the issues still to be defined is the best dosing schedule. In this study, we opted for an intensive therapeutic regimen (weekly) with up to eight infiltrations, which despite having produced a satisfactory cure rate, may be considered unattractive, requiring a large number of patient visits and an extended treatment time. Likewise, the maximum limit of drug per infiltration in order to avoid the already recognised antimony toxicity, observed with the intravenous or intramuscular route, is also not established. Considering the lack of available pharmacokinetic data for antimony administered by intralesional route, we opted for a more conservative approach and limited the maximum daily dose to that recommended for parenteral use (three ampoules). And finally, the small sample size (patients in each subgroup of interest, it means, patients with different number, size, location and morphology of lesions) and the scarcity of the failure episodes prevented the analysis of factors associated with therapeutic failure by multivariate analysis. Thus, the safety of the intralesional approach in specific subgroups of patients will still require further studies to be established. Thus, even without resolving several of the issues raised by intralesional therapy, we believe that the standardisation of the therapeutic procedure and the outcomes of interest in this study, which are in line with the current methodological recommendations for conducting clinical trials on CL^7^, make these results useful if they are added to others to support future and, ideally, multicentre studies.

Briefly, except for local events at the site of infiltration and the two hypersensitivity reactions, that alert to the need for strict surveillance, no specific organ systemic toxicity was observed in this study, which reinforces the safety of the approach and its feasibility specially in the context of a deficient laboratory infrastructure. This probably would be the advantage of intralesional antimony therapy: the reduction of well-known serious adverse events of antimony derivatives, which are the main cause of mortality observed among patients suffering from cutaneous leishmaniasis^22^. A detailed description of the clinical and laboratory adverse events observed during this phase II study will be presented in another manuscript together with pharmacokinetic data shortly.

It is clear that no single effective treatment for all CL cases is available and may never be achieved. The challenge is to identify the most appropriate treatment for each patient. Our findings suggest that the intralesional infiltration is effective at least for a selected group of patients with a limited number of cutaneous lesions. These observations certainly do not end the discussion about the optimal regimen for the MA intralesional infiltration therapy, nor do they establish whether the intralesional approach is superior or equivalent to systemic treatment. Apart from methodological limitations, issues related to efficacy in CL studies are difficult to tackle because of the many factors such as the intrinsic and acquired variations in the sensitivity of the different *Leishmania* species, host factors such as immunity, age of the lesions treated, drug toxicity, co-infection and compliance^18^. This work should contribute to the designing of new controlled trials in the future to support the incorporation of an ideal regimen based on MA intralesional infiltration that can ultimately contribute to the reduction of morbidity and mortality related to the leishmaniasis therapy.
